# Late normal tissue response in the rat spinal cord after carbon ion irradiation

**DOI:** 10.1186/s13014-017-0950-5

**Published:** 2018-01-11

**Authors:** Maria Saager, Peter Peschke, Thomas Welzel, Lifi Huang, Stephan Brons, Rebecca Grün, Michael Scholz, Jürgen Debus, Christian P. Karger

**Affiliations:** 1 Department of Radiation Oncology, University Hospital of Heidelberg, Heidelberg, Germany; 2 Department of Medical Physics in Radiation Oncology (E040), German Cancer Research Center (DKFZ), Im Neuenheimer Feld 280, 69120 Heidelberg, Germany; 3 National Center for Radiation Research in Oncology (NCRO), Heidelberg Institute for Radiation Oncology (HIRO), Heidelberg, Germany; 4 Clinical Cooperation Unit Molecular Radiooncology, German Cancer Research Center (DKFZ), Heidelberg, Germany; 5 Heidelberg Ion Beam Therapy Center (HIT), Heidelberg, Germany; 6 Department of Biophysics, Helmholtz Center for Heavy Ion Research (GSI), Darmstadt, Germany

**Keywords:** Carbon ion therapy, Relative biological effectiveness (RBE), Rat spinal cord, Magnetic resonance imaging, Radiation induced myelopathy

## Abstract

**Background:**

The present work summarizes the research activities on radiation-induced late effects in the rat spinal cord carried out within the “clinical research group ion beam therapy” funded by the German Research Foundation (DFG, KFO 214).

**Methods and materials:**

Dose–response curves for the endpoint radiation-induced myelopathy were determined at 6 different positions (LET 16–99 keV/μm) within a 6 cm spread-out Bragg peak using either 1, 2 or 6 fractions of carbon ions. Based on the tolerance dose TD_50_ of carbon ions and photons, the relative biological effectiveness (RBE) was determined and compared with predictions of the local effect model (LEM I and IV). Within a longitudinal magnetic resonance imaging (MRI)-based study the temporal development of radiation-induced changes in the spinal cord was characterized. To test the protective potential of the ACE (angiotensin converting enzyme)-inhibitor ramipril™, an additional dose–response experiment was performed.

**Results:**

The RBE-values increased with LET and the increase was found to be larger for smaller fractional doses. Benchmarking the RBE-values as predicted by LEM I and LEM IV with the measured data revealed that LEM IV is more accurate in the high-LET, while LEM I is more accurate in the low-LET region. Characterization of the temporal development of radiation-induced changes with MRI demonstrated a shorter latency time for carbon ions, reflected on the histological level by an increased vessel perforation after carbon ion as compared to photon irradiations. For the ACE-inhibitor ramipril™, a mitigative rather than protective effect was found.

**Conclusions:**

This comprehensive study established a large and consistent RBE data base for late effects in the rat spinal cord after carbon ion irradiation which will be further extended in ongoing studies. Using MRI, an extensive characterization of the temporal development of radiation-induced alterations was obtained. The reduced latency time for carbon ions is expected to originate from a dynamic interaction of various complex pathological processes. A dominant observation after carbon ion irradiation was an increase in vessel perforation preferentially in the white matter. To enable a targeted pharmacological intervention more details of the molecular pathways, responsible for the development of radiation-induced myelopathy are required.

## Background

Carbon ion therapy is increasingly applied in patients with skull base tumors [1, 2]. Although the clinical outcome is quite promising [3–5], a major limitation originates from the limited knowledge of the tolerance doses for late normal tissue reactions in the central nervous system (CNS), which mainly originates from the increased relative biological effectiveness (RBE) of carbon ions as compared to photons. As a result, radiation doses to the tumor and normal tissue are assessed in terms of RBE-weighted rather than absorbed dose [6]. The RBE, however, is a complex quantity and depends critically on the linear energy transfer (LET), on the fractional dose as well as on biological parameters and the considered biological endpoint. In clinical practice, the RBE is predicted quantitatively by biophysical models, such as the local effect model (LEM) [7], and this prediction includes significant uncertainties. Besides clinical investigations, experimental studies in animals have been performed to validate these RBE-models and to depict differences in the development of late CNS reactions between high- and low-LET irradiations.

The RBE of carbon ions in the rat spinal cord was evaluated for the endpoint radiation-induced myelopathy in previous studies [8–11], however, only one data set examined the dependence of the RBE on dose and LET [8, 9]. In those dose–response studies, irradiations of the spinal cord were performed in the entrance region and in the middle of a 1 cm spread-out Bragg peak (SOBP) using different fractionation schemes. Comparison of the results with predictions of the clinically used LEM I showed a significant underestimation of the RBE in the SOBP and deviations in the functional dependence on dose in the entrance region. These findings gave rise to further developments and resulted in the more recent version LEM IV [12], which is, however, not yet applied in patients. Since these early studies only covered two extreme LET-conditions, a systematic in vivo evaluation of the accuracy of the two model versions was not possible. Furthermore, although some early histological investigations to decipher radiation-induced myelopathy after carbon ion irradiation exist [13], no systematic studies on the temporal development and no correlation with findings in clinically relevant imaging modalities, such as magnetic resonance imaging (MRI), is presently available.

Within the translationally oriented clinical research group KFO 214 on heavy ion therapy, funded by the German Research Foundation (DFG), the radiation response of the rat spinal cord was extensively investigated. This contribution gives a brief summary of previously published data [14–16] in terms of dose–response curves for the endpoint radiation-induced myelopathy. Additionally, preliminary results of project-related, unpublished studies are presented including an MRI- and histology-based study to examine the temporal development of myelopathy. To protect the spinal cord from radiation-induced damage, the impact of an ACE- (angiotensin-converting-enzyme) inhibitor was tested.

## Methods and materials

### Animals and anesthesia

For the described studies, a total of 597 young adult female Sprague–Dawley (SD) rats (Charles River, Sulzfeld, Germany) were used. Animals were kept under standard conditions at the German Cancer Research Center (DKFZ) animal laboratory facility. For irradiations, rats received gaseous anesthesia with a mixture of 4% Sevoflurane (Abbott, Wiesbaden, Germany) and 2 l/min oxygen, whereas for the MRI-measurements 2.5 Vol% Isoflurane (Abbott, Wiesbaden, Germany) in 1.5 l/min oxygen was used. All experiments were approved by the governmental review committee on animal care (35–9185.81/G62–08, G117/13, G34/13).

### Follow-up and biological endpoint

After irradiation, animals were monitored once a week for general health condition and weight. Paresis grade II is defined as neurological symptoms by regular dragging of the foot with palmar flexion or dragging of extended foreleg [17]. A preliminary stage is paresis grade I meaning that the rat shows obvious neurological detractions but the animal is still able to use its forelegs.

The biological endpoint was defined as “radiation-induced myelopathy (paresis grade II) within 300 days”. Animals showing this endpoint were scored as responder, sacrificed and the spinal cord was processed for histological examinations.

### Dose–response studies

Details of the experimental setup has been described previously [14] and only a brief summary is given here. The rat cervical spinal cord (segments C1–6, field size 10 × 15 mm^2^) was irradiated at 6 different positions (35, 65, 80, 100, 120 and 127 mm) of a 6 cm spread-out Bragg peak (SOBP, range 70–130 mm water-equivalent depth) corresponding to a dose-averaged linear energy transfer (LET) of 16–99 keV/μm. The range of the ions was adjusted using appropriate polymethyl-methacrylate (PMMA)-boli placed in front of the animals. Irradiations were performed in groups of 5 animals with increasing dose levels using either 1 or 2 fractions (Fx) to cover 0–100% response probability. Animal numbers were selected to determine TD_50_ (dose at 50% probability of paresis grade II) with a standard error of about 0.5 Gy. Irradiations were performed under identical conditions either at the Helmholtz Center for Heavy Ion Research (GSI, 100 mm mid-position), or (after beam time became available) at the Heidelberg Heavy Ion Therapy Center (HIT, all other positions) using the active raster scanning technique [18]. The presented results for 1 and 2 Fx included a total of 464 irradiated rats as well as 10 sham treated controls.

For each fractionation schedule and each position of the spinal cord within the SOBP, a dose–response curve was determined by performing a maximum-likelihood fit of the logistic dose–response model to the actuarial response rates (technical details, see [14, 15]). Based on the TD_50_-values of photons [8, 9] and carbon ions, the RBE was calculated. The experimental RBE was compared to model predictions using the version I and IV of local effect model (LEM) [7, 12]. RBE-calculations with the LEM were performed with the treatment planning system TRiP (Treatment Planning for Particles [19]) for the experimentally obtained TD_50_-values.

### MRI-based longitudinal study

To investigate the temporal development of radiation-induced myelopathy, 24 irradiated animals and 7 sham-treated controls were included in an MR-based longitudinal study. Irradiated animals received 6 Fx of either carbon ions (center of 1 cm SOBP; LET: 91 keV/μm (range, 80–104 keV/μm)) or 6 MV photons using approximately isoeffective total doses of 23 Gy (RBE) or 61 Gy, respectively. Based on our previous study [8], these doses were known to cause radiation-induced myelopathy in all animals.

For imaging, a 1.5 T MRI scanner (Symphony, Siemens, Erlangen) in combination with an in-house made radio-frequency coil was used. To record the initial state, rats were imaged prior to irradiation. After irradiation, rats were monitored monthly and as soon as morphological alterations in the MR-images occurred, the measurement intervals were reduced.

MRI measurements included a T2-weighted sequence (TE 109 ms, TR 4000 ms, FOV 40 mm) to detect edema. To prove the onset of a blood-spinal cord barrier (BSCB) disruption a T1-weighted sequence (TE 14 ms, TR 600 ms, FOV 46 mm) in combination with contrast agent application (0.2 mmol/kg, Magnevist®, Bayer, Leverkusen) was used. In addition, a T1-weighted dynamic contrast-enhanced (DCE) MR sequence (TE 1.75 ms, TR 373 ms, FOV 150 mm) was used to study radiation-induced alterations in blood perfusion. DCE-measurements were evaluated using a pharmacokinetic model [20, 21] allowing the determination of the relative plasma volume, v_p_, the relative interstitial volume, v_e_ and the volume transfer coefficient K_trans_.

### Histology

Animals reaching the endpoint paresis grade II were perfused with a mixture of 4% paraformaldehyde (PFA) in 0.015 M phosphate buffered saline. The cervical spinal cord C1–6 was dissected out and postfixed overnight. Cryosections of 8 μm thickness were used for a general staining with hemalum/eosin (HE) in combination with Luxol fast blue [22]. Luxol fast blue was used to examine qualitatively the extent of demyelination since the dye attaches to the lipoproteins of the myelin. A reduced signal is assigned with affected areas.

To study the degree of blood vessel perforation, extravasated serum albumin was immunohistochemically visualized. For this, paraffin sections of 8 μm thickness were deparaffinized and rehydrated. Endogenous peroxidase activity was blocked with 3% H_2_O_2._ To unmask antigen sites, an antigen retrieval with sodium citrate buffer (pH 6) was performed. Sections were then incubated overnight at 4 °C with the primary antibody against albumin (Acris, 1:6000 diluted in 3% bovine serum albumin) followed by incubation with the secondary antibody (Abcam, 1:500, horse raddish peroxidase). 3,3′-diaminobenzidine was used as chromogen. Afterwards the sections were counterstained with Nissl and evaluated by light microscopy.

### Radioprotectiva study

The protective influence of the ACE-inhibitor ramipril™ was investigated in a four-armed dose–response experiment using a total of 88 animals and four sham-treated controls. Animals were irradiated with single doses of carbon ions (center of 6 cm SOBP; LET: 45 keV/μm) or 6 MV photons. 4 animals per dose group with increasing dose levels were used to cover 0–100% response probability. Each modality includes an experimental arm with and without ramipril™ administration. The ACE-inhibitor was given immediately after irradiation (2 mg/kg/day) via their drinking water (ad libitum) during the full observation time of 300 days.

## Results

The irradiation procedure, MRI follow-up and ACE-inhibitor intake was well tolerated by all animals. Rats which had to be excluded during follow-up due to spontaneous development of mammary carcinomas or death due to unknown reasons were considered by an actuarial approach.

### Dose–response studies

**Fig. 1:**
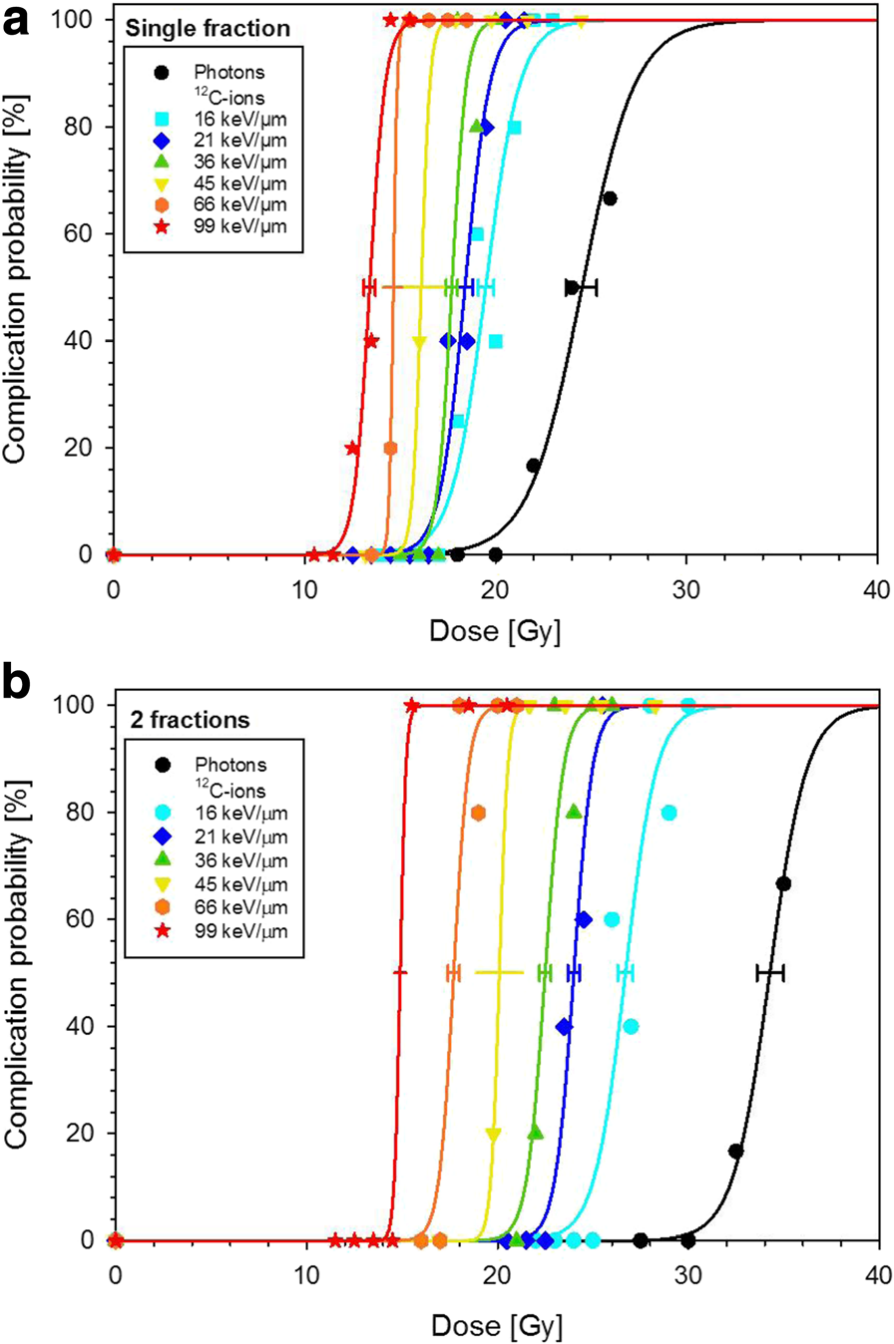
Dose–response curves for the endpoint paresis grade II after irradiation of the spinal cord with either single (**a**) or split (**b**) doses of carbon ions or photons at 6 different positions within a 6 cm SOBP

**Fig. 2:**
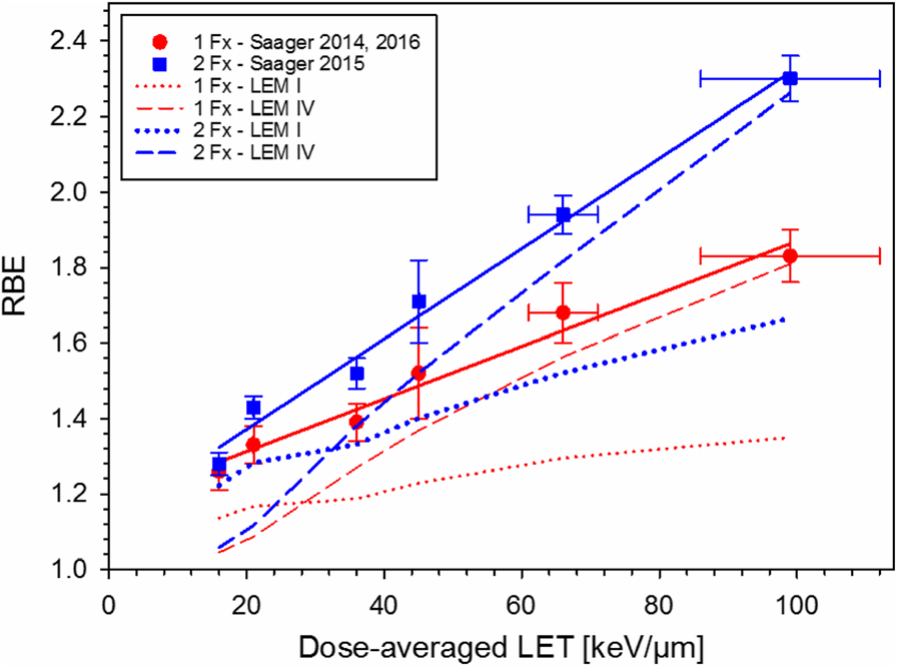
RBE-dependence on LET for single and split doses. In addition, RBE-values predicted by LEM I and LEM IV are displayed

Figure 1 summarizes the dose–response curves obtained at the 6 positions within the SOBP after one and two fractions of carbon ions. The corresponding TD_50_-values decreased significantly with increasing LET and increased with increasing fraction number, i.e. decreasing fractional dose. Figure 2 displays the resulting LET-dependence of the RBE after single and split doses. It was found that the RBE increases much stronger after 2 fractions than after single fractions. Comparing the measured RBE-values with predictions of the LEM revealed that LEM IV better predicts this stronger increase, and in general provides a much better description in the high-LET region (30–100 keV/μm) of the SOBP while LEM I is more accurate in the low-LET region (~20 keV/μm) of the plateau.

### MRI-based longitudinal study

**Fig. 3:**
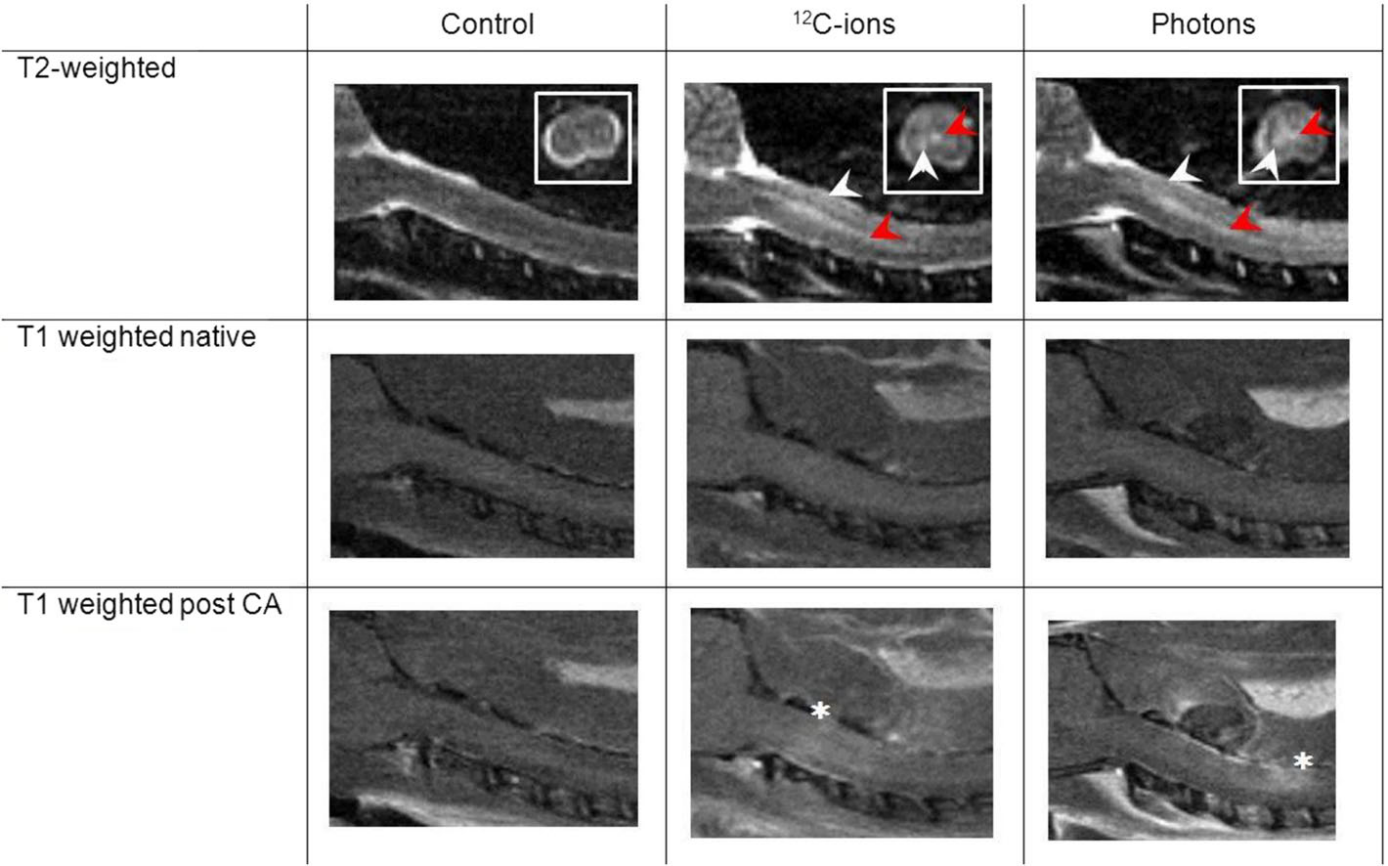
Representative MR-images for the biological endpoint paresis grade II after carbon ion (^12^C–ion) and photon irradiation compared to an untreated control. The symptomatic animals show an edema (white arrowheads) and *canalis centralis* dilatation (red arrowhead) in the T2-weighted images as well as contrast agent (CA) accumulation in the T1-weighted images (lowest row, white asterisks)

MRI measurements after carbon ion and photon irradiation revealed the same morphological alterations in the MR images ranging from the development of edema, syrinx (dilatation of the *canalis centralis*) and contrast agent accumulation up to the final development of the radiation-induced myelopathy (Fig. 3). The latency time until development of paresis grade II, however, was significantly shorter for carbon ions (136 ± 10 d) than for photons (211 ± 20 d). Evaluation of the DCE-measurements exhibited a continuous increase of the parameters v_e_ and K_trans_ with increasing damage of the BSCB, however, no significant differences between carbon ion and photon irradiation was found, except for the shorter latency time. No significant changes were found for the parameter v_p_.

### Histology

**Fig. 4:**
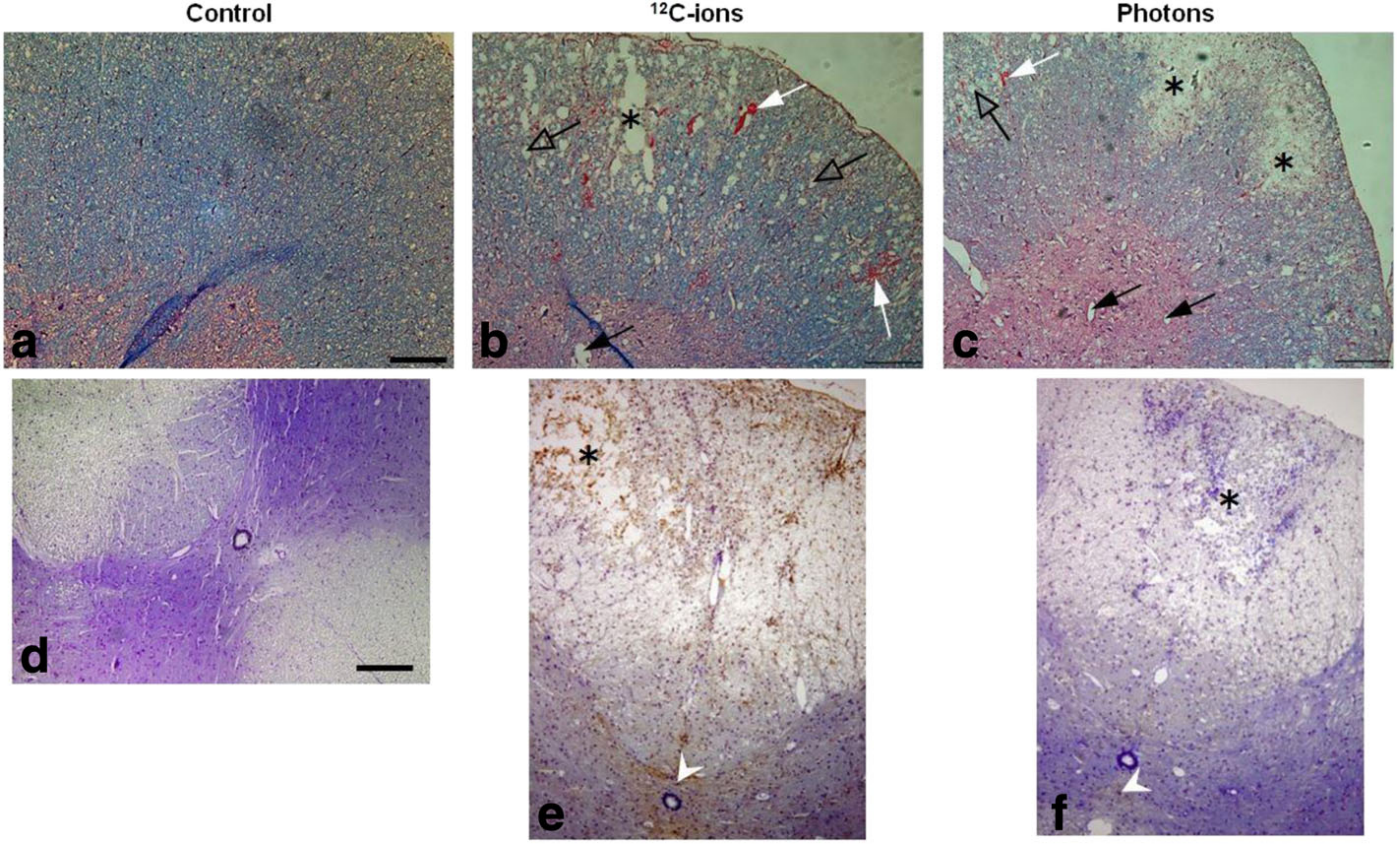
Histological sections representative for the biological endpoint paresis grade II. Cryosections stained with hemalum/eosin in combination with Luxol fast blue (**a**-**c**). A clear structural decline in the white matter represented by necrosis (asterisk) and vacuolization (open arrows) as well as hemorrhages (white arrows) and dilated blood vessels (closed black arrows) can be seen (**b**, **c**). Paraffin sections for detection of albumin extravasation (brown precipitation) combined with Nissl staining (**d**-**f**). Albumin leaks predominately in the area where structural decline of white matter occurs (black asterisks) and around the *canalis centralis* (white arrow heads). The leakage is more intense after carbon ion (**e**) than photon irradiation (**f**) (scale bar 200 μm)

After carbon ion as well as after photon irradiation histological examinations of the endpoint paresis grade II revealed a comparable extent of tissue damage (Fig. 4). Compared to the unirradiated control, a structural decline in terms of white matter vacuolization, necrosis, blood vessel dilatation and disruption was found in the posterior and lateral part for both radiation modalities. A clear demyelination represented by the loss of luxol fast blue staining has been seen after photon irradiation (Fig. 4c). The blood vessels in the grey matter were dilated and perforated whereas the overall structure remained visually intact. However, a larger extent of blood vessel perforation was found after carbon ion than after photon irradiation. The albumin extravasation, represented by a brown precipitation, was more intense after carbon ion irradiation, predominately in the dorsal part of the white matter and around the *canalis centralis* whereas after photon irradiation the albumin extravasation was found to be weaker in these areas (Fig. 4).

### Radioprotectiva study

No protective effect of ramipril™ for the development of radiation-induced myelopathy after carbon ion or photon irradiations was observed. However, a modality and dose dependent prolongation of latency time of 23 ± 8 d after carbon ion irradiation and 16 ± 3 d after photon irradiation was found.

## Discussion

Only very few studies on late effects in normal tissue are presently available [11, 13, 23, 24]. Radiation-induced myelopathy is a feared late side effect in the CNS, characterized by a long symptom-free latency period followed by a sudden occurrence of neurological symptoms. To prevent the development of these severe complications, specific tolerance doses have to be respected and due to the uncertainty in the knowledge of the RBE, this is associated with significantly larger uncertainties for carbon ions than for photons.

To investigate the accuracy of RBE-predictions by the LEM, a large-scale dose–response study in the rat spinal cord has been performed. This animal model is well established for the investigation of late effects in the CNS and has been previously used to study the effectiveness of different beam modalities [25–30]. Especially, it has been shown that the response of the spinal cord is independent of the irradiated volume for field lengths above 8 mm [31, 32]. The model is also well-suited to study the temporal development of radiation-induced myelopathy in MRI as well as on the histological level. This study currently presents the largest and most systematic data base.

### Dose–response studies

The rat spinal cord was used to characterize the RBE-variation along the central axis of a 6 cm SOBP for different fractionation schedules. The details of these studies have been published previously [14–16]. Detailed in vivo testing of the RBE-predictions of LEM I and IV as a function of LET and fractional dose revealed that the RBE in the high-LET region is better described by LEM IV while the predictions of LEM I are more accurate in the low-LET region. It has to be noted, however, that this result refers to relatively high fractional doses. An additional dose–response study with 6 Fx is currently under evaluation and will allow to extend the benchmarking of the LEM also towards lower doses per fraction. Together with the presented results, this study will allow to estimate the α/β-value, which represents the extent of tissue regeneration in fractionated treatments. Preliminary results based on the single and split dose studies suggest an increase of α/β with increasing LET, indicating a decreasing impact of fractionation for increasing LET. For a more reliable estimation, however, the 6 Fx study has to be included. It has to be emphasized that the benchmarking of RBE-models is not restricted to the LEM. Currently, tests are extended to the Microdosimetric Kinetic Model (MKM) which is used for carbon ion therapy at National Institute of Radiological Science (NIRS, [33, 34]).

### MRI-based longitudinal study

The MRI-based longitudinal study enables a non-invasive investigation of occurring radiation-induced effects during the symptom free latency time. We found a fixed sequence of alterations in the images. Comparing the carbon ion and photon irradiations at isoeffective doses with respect to the endpoint paresis grade II, the same morphological changes were found and the only difference was a shorter latency time after carbon ion irradiation. Main findings in MRI were presence of edema, syrinx, uptake of contrast agent due to the break-down of the BSCB and finally followed by paresis grade I and II. Once the edema occurred in an animal, it developed the deterministic sequence. These findings were also confirmed quantitatively by evaluation of the DCE-measurements, which showed that the increase of the extracellular volume, v_e_, and the contrast agent exchange rate, K_trans_, increased similarly for carbon ions and photons.

It appears likely that the shorter latency time after carbon ion irradiations originates from differential actions on the histological or molecular level and apparently, MRI at 1.5 T is not sensitive enough for the detection of such alterations. With respect to sensitivity, the small diameter of the rat spinal cord and the consequently occurring partial volume effects may also play a role. Using an MRI with higher field strength would in principle be an option to increase the sensitivity, yet, in the present study, this was logistically not possible due to the excessive number of measurements, which had to be performed on a short term notice during the period where neurological symptoms appear within a rapid time sequence.

Despite of these limitations, this study provides the first extensive temporal characterization of the development of radiation-induced myelopathy after irradiation with carbon ions and photons in MRI and in an ongoing MRI-based histological study, tissue samples at different time points after irradiation as well as at the occurrence of the different endpoints in MRI are acquired. By investigating these samples on the histological and molecular level, more detailed information on the underlying mechanistic processes is expected.

### Molecular mechanisms and inhibition

Currently, it is not clear in detail whether the target structures of irradiation in the spinal cord are the neurons or the blood vessels. Therefore, many attempts have been made to evaluate the effects of ionizing radiation to the neuronal [22, 35–37] and the vascular proportion [11, 24, 38–41] supporting nowadays the view that endothelial cells are the main target structure [42–44].

At the endpoint paresis grade II, histological examinations revealed a comparable breakdown of the tissue structure for both radiation modalities; however, the increase of blood vessel permeability was much higher after carbon ion irradiation. This finding is in contrast to results of the DCE evaluation, where no difference was seen at the same endpoint.

It has to be noted, however, that increased permeability of the BSCB was detected with albumin, which presents a much larger molecule than MRI contrast agent Gd-DTPA (66 vs. 0.5 kDa). The discrepancy between the results of MRI and histological analysis could therefore be explained by a different extent of perforation for the two irradiation modalities. While the higher ionization density of carbon ions introduces more complex, non-reparable DNA-damage, which leads to intense blood vessel perforation and thus to an increased permeability for Gd-DTPA as well as for albumin, photons exhibit a low ionization density which induces better reparable DNA-damage and leads only to small vessel perforations and thus to an increased permeability for Gd-DTPA but much less for albumin. To clarify this, additional histological investigations with smaller molecular markers are required.

Besides vascular changes, also a profound damage of the neuronal structures was observed. Luxol fast blue staining shows a clear decrease of the myelin basic protein at the biological endpoint paresis grade II. To assess the relative importance of vascular and neuronal damage, a detailed investigation of the temporal development of both structures on the histological and molecular level will be performed within the ongoing MRI-based histological study.

Detailed knowledge of the mechanistic processes may enable targeted pharmacological interventions with the aim of protecting the normal central nervous system tissue after irradiation. First attempts along this direction have already been described in the literature [45–48] using ACE-inhibitors. Within a pilot trial, we used the ACE-inhibitor ramipril™ to test the impact on radiation-induced myelopathy after carbon ion and photon irradiation. The rationale for using this drug are manifold: ramipril™ has been shown to exhibit mitigative properties on optic neuropathy [47, 49]. In addition, with regard to the central nervous system, the drug is able to cross the blood-spinal cord barrier [50], does not reveal protective effects on tumors [51] and is already used to treat hypertension in patients. Our results showed that myelopathy could not be prevented, however a prolongation of latency time was achieved, which indicates that ramipril™ has a mitigative effect in the rat spinal cord. Identification of the underlying pathological pathways leading to radiation-induced side effects would facilitate the application of appropriate protective drugs and, if successfully realized, could allow elevating the tumor dose without harming the surrounding normal tissue.

## Conclusion

Within this study, a large data base on RBE for late effects in CNS tissue of the rat after carbon ion irradiation was established and used for benchmarking the functional dependencies of the RBE on LET and dose as predicted by LEM I and LEM IV. According to this comparison, LEM IV better describes the measured data in the high-LET region while LEM I predictions are more accurate in the low-LET region. Ongoing studies will extend this database further. Using MRI, an extensive characterization of the temporal development of radiation-induced alterations in the rat spinal cord was obtained. The main result was a shorter latency time for carbon ions than for photons. This finding is expected to originate from complex pathological pathways on the molecular level, which needs further investigations. This hypothesis is supported by histological investigations, where an increased vessel perforation, associated with a differential pattern of permeability, was found after carbon ion as compared to photon irradiations. For the ACE-inhibitor ramipril™, a mitigative rather than protective effect was found, however, the design of targeted protective drugs requires more detailed knowledge on the molecular pathways during the pathogenesis of radiation-induced myelopathy.

## Abbreviations

^12^C–ion: Carbon ion
ACE: Angiotensin-converting-enzyme
BSCB: Blood-spinal cord barrier
CNS: Central nervous system
DCE: Dynamic contrast enhanced
FOV: Field of view
Gd-DTPA: Gadolinium diethylenetriaminepentacetate
LEM: Local effect model
LET: Linear energy transfer
MKM: Microdosimetric kinetic model
MRI: Magnetic resonance imaging
NIRS: National Institute of Radiological Science
RBE: Relative biological effectiveness
SD: Sprague Dawley
SOBP: Spread-out Bragg Peak
TD: Tolerance dose
TE: Echo time
TR: Repetition time
TRiP: Treatment planning for particles
